# SRSF1 inhibition of HIV-1 gene expression

**DOI:** 10.18632/oncotarget.5125

**Published:** 2015-08-10

**Authors:** Sean Paz, Massimo Caputi

**Affiliations:** Charles E. Schmidt College of Medicine, Florida Atlantic University, Boca Raton, FL, USA

**Keywords:** SRSF1, HIV-1, Immunology and Microbiology Section, Immune response, Immunity

The treatment of HIV-1 infected patients is mostly based on compounds that directly target the activity of proteins coded by the virus. However, the viral genome mutates at a rapid rate, generating drug resistant strains, hence the need for novel drugs to outpace the adaptive mechanisms of the virus. Since cellular factors, which are required for the efficient expression of the HIV-1 genome, do not mutate in response to drugs and have limited polymorphisms in the population their identification and study may provide therapeutic targets that are not easily circumvented by the high mutation rate of the virus.

Genomic screenings utilizing siRNA libraries have helped in the identification of several cellular proteins required for HIV-1 replication and infectivity [1]. The factors isolated belong to a variety of functionally diverse protein families and a number of strategies aimed at limiting their activity in the viral life cycle are being developed. Nevertheless, inhibiting the activity of cellular proteins by small chemical compounds may negatively affect key cellular processes while silencing cellular factors by interfering RNAs is often inefficient *in-vivo*. An alternative approach is the screening of complementary DNA libraries for genes that inhibit viral replication. In recent work we screened a RNA Binding Protein (RBP) expression library to identify cellular proteins that modulate the replication of HIV-1 [2]. Among the factors identified, SRSF1, a well-characterized splicing factor, displayed a strong activity as an inhibitor of viral transcription. SRSF1 is a member of the serine/arginine (SR) proteins family. SR proteins are widely expressed in eukaryotes and regulate gene expression by controlling the assembly of the splicing machinery, integrating multiple steps in RNA metabolism and modulating RNA Polymerase II (RNAPII) activity [3].

The cellular machineries regulating the transcription and processing of eukaryotic RNAs are intimately coupled. RBPs have been shown to regulate both transcriptional and post-transcriptional events by connecting the transcription complex to the nascent RNA. The replication of the integrated HIV-1 genome is regulated by a combination of host and viral factors. RNAPII and a combination of basal and promoter specific factors assemble onto the HIV promoter; while the viral protein Tat stimulates transcription by binding a structured RNA element (TAR: trans-activating response element), located in the nascent transcript, and modifying the composition of the active RNAPII complex. Approximately 50% of the viral transcripts leave the nucleus without being spliced, code for the Gag and Gag-Pol polyprotein and are packaged within the nascent virions as viral genome. The remaining transcripts undergo a series of splicing events to generate over 40 mRNA isoforms, which code for the remaining viral protein [4]. SRSF1 down-regulates expression of the viral genome by competing with Tat for its binding onto TAR [2]. Additionally, it regulates splicing by binding short sequences throughout the viral messenger and modulating the usage of several splice sites [4]. Given the multiple SRSF1 binding sites present throughout the viral genome and the complex splicing pattern of the viral mRNAs it is not surprising that changes in SRSF1 expression can severely affect viral replication. Our data indicate that over-expression of SRSF1 reduces replication of viral isolates of the B, C and D subtypes by a minimum of 100 folds in stable cell lines [5].

**Figure 1:**
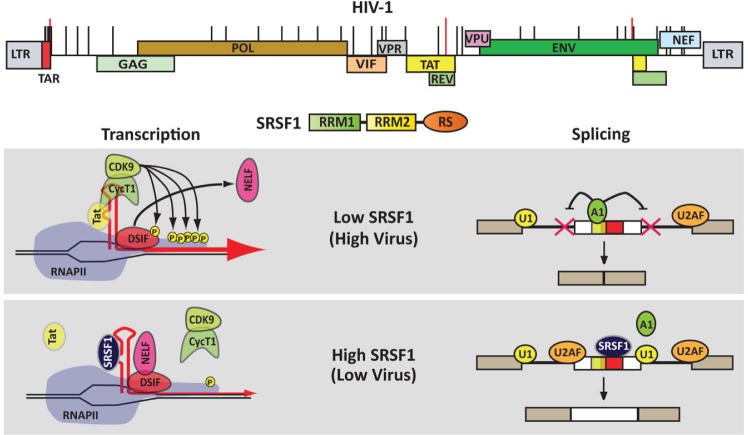
SRSF1 inhibits HIV replication Several SRSF1 binding sites have been experimentally characterized (red lines) and predicted (black lines) within the HIV-1 transcript. SRSF1 binding onto the viral transcript can impact both, transcription and splicing. Our data [2, 5] indicate that at a low SRSF1 concentration the viral protein Tat binds TAR and increases viral transcription by modifying the composition of the transcription complex and inducing phosphorylation of RNAPII. At high concentrations SRSF1 competes with Tat for binding onto TAR, which results in a reduction of viral transcription. Similarly, splicing of the viral mRNA is modulated by cellular factors that bind sequences in proximity of the splice sites and regulate the recruitment of spliceosomal components. SRSF1 can compete with such regulatory factors by binding onto overlapping sequences within the target transcript.

A major drawback in utilizing cellular proteins as therapeutic targets is the risk of impacting cellular metabolism and viability. SRSF1 is composed of two RNA-binding domains (RBDs) and an RS (arginine/serine-rich) domain, which is required for proteinprotein interaction but does not appear to affect the RNA binding specificity of the protein. Since SRSF1's role in HIV replication appears to be based solely on its RNA binding properties [2, 5], in order to minimize the effect on multiple cellular mechanisms we tested deletion clones that express the protein RBDs but lack the RS domain. The deletion mutants exhibited an inhibition of viral replication higher than the full-length protein (2000 vs 100 folds), while having a minimal impact on cell viability [5]. The enhanced antiviral activity observed with the deletion mutants may be due to the role played by the RS domain in promoting the association of SRSF1 with spliceosomal complexes and other cellular structures. In the absence of the RS domain the protein is not sequestered within these complexes and it is available to bind the viral transcripts.

To properly assess the therapeutic potential of SRSF1 it is necessary to determine its antiviral properties in a physiologically relevant infection model such as primary CD4+ T cells, which are the main viral target. Unfortunately, the majority of the approaches utilized to transduce leukocytes are based on lentiviral or electroporation delivery systems. These methodologies have severe limitations in both *in-vitro* and *in-vivo* systems and do not yield a homogenous transduction of the cells treated. To overcome these drawbacks alternative approaches can be developed by utilizing chimeric proteins containing the SRSF1 RBDs conjugated to cell penetrating peptides (CPPs). CPPs can cross highly selective barriers, like the intestinal wall or the blood-brain barrier, and deliver the conjugated protein in difficult to transfect primary cells and animal models making them a more suitable system to deliver the SRSF1 RBDs to CD4+ T cells *in-vitro* and *in-vivo* [6].
